# Responses to land cover and grassland management vary across life‐history stages for a grassland specialist

**DOI:** 10.1002/ece3.6805

**Published:** 2020-10-29

**Authors:** Michael A. Hardy, Matthew S. Broadway, Christopher D. Pollentier, Volker C. Radeloff, Jason D. Riddle, Scott D. Hull, Benjamin Zuckerberg

**Affiliations:** ^1^ Department of Forest and Wildlife Ecology University of Wisconsin‐Madison Madison WI USA; ^2^ College of Natural Resources University of Wisconsin‐Stevens Point Stevens Point WI USA; ^3^ Office of Applied Science Wisconsin Department of Natural Resources Madison WI USA; ^4^ Present address: Biogeographic Data Branch California Department of Fish & Wildlife Sacramento CA USA; ^5^ Present address: Indiana Department of Natural Resources Bloomington IN USA

**Keywords:** brood survival, grassland bird, grouse, habitat selection, hen survival, nest survival, prairie chicken, *Tympanuchus*

## Abstract

Grassland birds have exhibited dramatic and widespread declines since the mid‐20th century. Greater prairie chickens (*Tympanuchus cupido pinnatus*) are considered an umbrella species for grassland conservation and are frequent targets of management, but their responses to land use and management can be quite variable. We used data collected during 2007–2009 and 2014–2015 to investigate effects of land use and grassland management practices on habitat selection and survival rates of greater prairie chickens in central Wisconsin, USA. We examined habitat, nest‐site, and brood‐rearing site selection by hens and modeled effects of land cover and management on survival rates of hens, nests, and broods. Prairie chickens consistently selected grassland over other cover types, but selection or avoidance of management practices varied among life‐history stages. Hen, nest, and brood survival rates were influenced by different land cover types and management practices. At the landscape scale, hens selected areas where brush and trees had been removed during the previous year, which increased hen survival. Hens selected nest sites in hay fields and brood‐rearing sites in burned areas, but prescribed fire had a negative influence on hen survival. Brood survival rates were positively associated with grazing and were highest when home ranges contained ≈15%–20% shrub/tree cover. The effects of landscape composition on nest survival were ambiguous. Collectively, our results highlight the importance of evaluating responses to management efforts across a range of life‐history stages and suggest that a variety of management practices are likely necessary to provide structurally heterogeneous, high‐quality habitat for greater prairie chickens. Brush and tree removal, grazing, hay cultivation, and prescribed fire may be especially beneficial for prairie chickens in central Wisconsin, but trade‐offs among life‐history stages and the timing of management practices must be considered carefully.

## Graphical abstract

Greater prairie chickens (*Tympanuchus cupido pinnatus*) are considered an umbrella species for grassland conservation and are frequent targets of management, but their responses to land use and management can be quite variable. We examined habitat, nest‐site, and brood‐rearing site selection by hens and modeled effects of land cover and management on hen, nest, and brood survival. Collectively, our results highlight the importance of evaluating responses to management efforts across a range of life‐history stages and suggest that a variety of management practices are likely necessary to provide structurally heterogeneous, high‐quality habitat for greater prairie chickens.

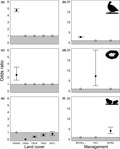

## 1 INTRODUCTION

Habitat loss and fragmentation pose substantial threats to many species and communities across the majority of Earth's biomes (Fahrig, 1997, 2003; IUCN, 2018). However, not all biomes have been equally affected. Of all major terrestrial biomes, temperate grasslands, shrublands, and savannas exhibited the third highest rate of loss (45.8%) and smallest representation within protected areas (4.6%; Hoekstra et al., 2005). Consequently, these systems may face the greatest risk of biome‐wide biodiversity loss. In North America, extensive loss, fragmentation, and conversion to agricultural land use following European settlement has led to precipitous declines in both quantity and quality of native grasslands (Samson et al., 1998). Remaining grasslands are among the most extensively altered ecosystems (Askins et al., 2007) and currently exist largely as remnant patches of “surrogate” grassland habitat such as hay fields, pasture, and fallow fields (Sample et al., 2003). Coincident with the loss and degradation of grassland systems, grassland bird populations have declined dramatically (Rosenberg et al., 2019; Sauer et al., 2017; Vickery & Herkert, 2001), more so than any other group of birds in North America (Knopf, 1994). Because patterns of land ownership can present formidable obstacles to securing additional parcels and aggregating remnant grasslands, natural resource managers frequently have limited options for conservation and are often forced to rely on intensive management of remnant patches on public lands.

Management practices, such as grazing, prescribed burning, mechanical brush removal, or mowing, can have profound effects on vegetative structure of grasslands, affecting grassland birds directly and indirectly. Henslow's Sparrows (*Ammodramus henslowii*), for example, were not encountered in patches that had experienced focal disturbance from grazing combined with burning within the past 12 months, but increased in abundance with time since disturbance (Fuhlendorf et al., 2006). Conversely, Upland Sandpipers (*Bartramia longicauda*), Killdeer (*Charadrius vociferus*), and Lark Sparrows (*Chondestes grammacus*) were most abundant in recently disturbed patches and their abundance declined as vegetation recovered (Fuhlendorf et al., 2006). Lesser prairie chickens (*Tympanuchus pallidicinctus*) avoided nesting in pastures that had been previously treated with herbicide (Johnson et al., 2004), presumably because spraying eliminated the shrub cover often selected by hens for nest sites (Hagen & Giesen, 2005). Lastly, in agricultural landscapes, hay or silage harvest can have dramatic effects on bird survival and reproductive success by reducing height and density of vegetation or causing direct mortality (Grüebler et al., 2008).

Additionally, effects of management practices differ not only among species, but also among life‐history stages of the same species. For example, adult Upland Sandpipers selected recently burned sites within their home ranges, but selected infrequently burned areas for nest sites, which increased nest survival (Sandercock et al., 2015). Moreover, management practices do not always have the same influence on habitat selection behavior and demographic rates: Grasshopper Sparrows (*A. savannarum*) were more abundant in grazed pastures compared to hayfields or Conservation Reserve Program grasslands, but nest survival showed the opposite pattern and was greatest in burned hayfields (Rahmig et al., 2009), highlighting an apparent disconnect between habitat selection and habitat quality. Given these trade‐offs, failure to consider both habitat selection and habitat quality might lead to false assessments of the effectiveness of management practices, and potentially even the creation of ecological traps (i.e., selection of low‐quality habitat; Battin, 2004). However, obtaining the data necessary to conduct a comprehensive assessment of habitat selection behavior and demographic responses across multiple life‐history stages is time‐ and resource‐intensive and such assessments remain comparatively rare.

**Figure 1:**
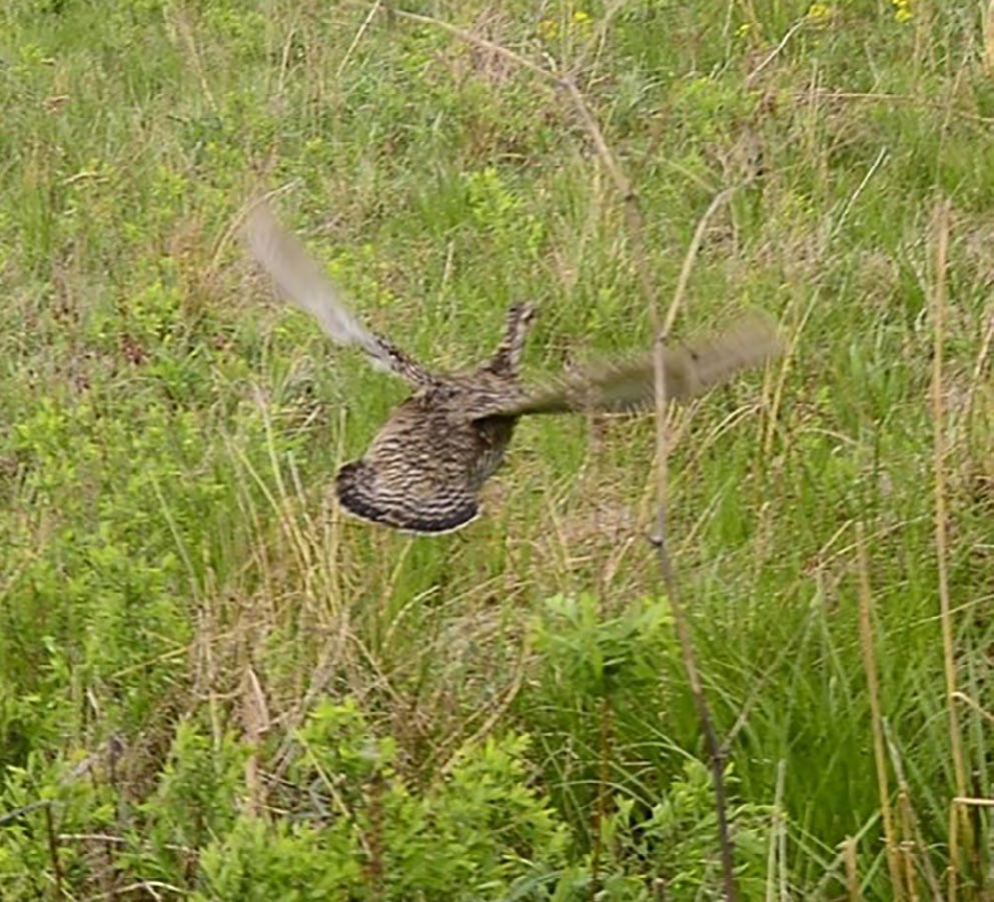
The greater prairie chicken (*Tympanuchus cupido pinnatus*) is a grassland specialist that has suffered from a dramatic long‐term decline in several parts of its range, including Wisconsin, USA, where it is a species of special concern and is a frequent target of management. Photo © M. S. Broadway, 2015

Greater prairie chickens (*T. cupido pinnatus,* hereafter “prairie chicken”; Figure 1), often considered an “umbrella” species for grassland conservation (Poiani et al., 2001), were once widespread and abundant throughout grasslands and oak savannas of central North America. However, like many other grassland birds, prairie chickens have declined dramatically since European settlement (Johnson et al., 2011). Although prairie chickens are still numerous and legally harvested in several states, they have been extirpated from much of their historic range and currently persist only as small, isolated populations in many regions (Johnson et al., 2011). Prairie chickens are considered area sensitive and require relatively large patches of open habitat (Hamerstrom et al., 1957; Johnson et al., 2011; Winter & Faaborg, 1999). Moreover, prairie chickens are sensitive to management practices and both behavioral and demographic responses to management have been documented in previous studies (Hovick et al., 2015; Patten et al., 2007; Winder et al., 2018).

Due to widespread population declines and sensitivity to land management, prairie chickens are a conservation priority in many states, including Wisconsin, USA (Hull et al., 2011; Wisconsin Department of Natural Resources, 1995). Prairie chickens have experienced a significant range contraction in Wisconsin and are currently state‐listed as threatened, with an estimated ≤1,000 birds remaining (Hardy et al., 2018). Because much of the available prairie chicken habitat is privately owned and remains in agricultural production, opportunities for public agencies or conservation organizations to acquire grasslands for prairie chicken management are limited. Consequently, conservation efforts are often restricted to intensive management of state‐owned parcels. Unfortunately, despite a long history of intensive management, including translocations (Bateson et al., 2014; Hardy et al., 2018; Hull et al., 2013), prairie chickens have continued to decline in Wisconsin, prompting questions regarding their responses to on‐the‐ground habitat management.

Here, we used data collected during 2007–2009 and 2014–2015 to evaluate the effects of land cover and management on habitat selection and survival of greater prairie chickens in Wisconsin. We quantified the effects of land cover and several common grassland management practices (e.g., grazing, burning, and haying) during three life‐history stages (adult, nest, and brood). Our study represents a major step forward in our understanding of the effects of management on prairie chickens near the northern extent of their range boundary. By focusing on multiple facets of habitat selection and demography for prairie chickens occupying highly modified landscapes, we present a comprehensive assessment of the effects of land cover and management practices on a vulnerable and declining grassland bird.

## 2 METHODS

### 2.1 Study area

**Figure 2:**
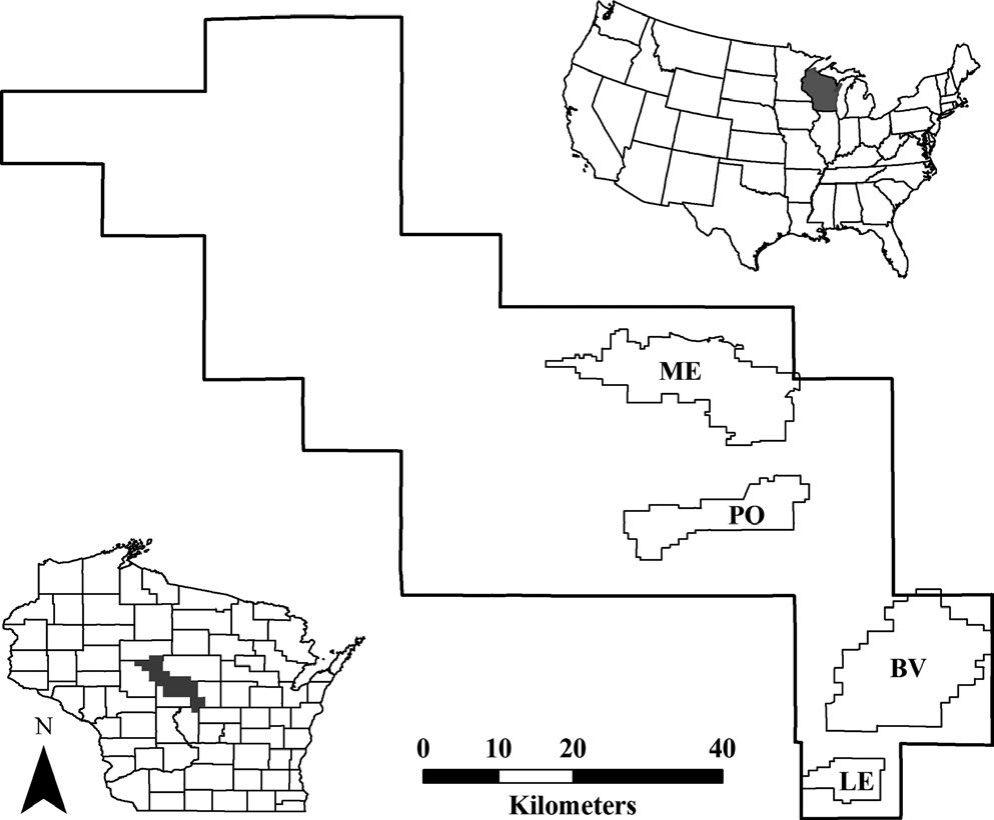
Location of four core sites (Buena Vista, BV; Paul Olson, PO; Leola, LE; Mead, ME) in the Central Wisconsin Grassland Conservation Area, Wisconsin, USA. Data used in this study were collected at BV, PO, and LE from 2007–2009 and 2014–2015

In Wisconsin, the largest remaining populations of prairie chickens occur in four relatively isolated wildlife areas in the Central Wisconsin Grassland Conservation Area (CWGCA): Buena Vista Marsh Wildlife Area, Paul J. Olson Wildlife Area, Leola Marsh Wildlife Area, and George W. Mead Wildlife Area (Wisconsin Department of Natural Resources, 2004; Figure 2). These core properties range in size from 3,394–18,975 ha and consist of a diverse mosaic of grasslands, wetlands, shrublands, forests, and various forms of agriculture (Niemuth, 2000). In recent decades, land use in the surrounding landscape has shifted from pasture and other less‐intensive forms of agriculture to irrigated center‐pivot and intensive row crop production (i.e., corn, soybeans, alfalfa, potatoes), and, to a lesser extent, cranberry cultivation. In 1968, ≈275 ha of private grassland had been converted to intensive center‐pivot irrigation (Hamerstrom & Hamerstrom, 1973), whereas ≥4,455 ha had been converted by 1998 (Anderson & Toepfer, 1999); since 2003, the prevalence of row crops has continued to increase, with a concurrent decline in grassland cover (M.A. Hardy, *unpublished data*). During the prairie chicken breeding season (i.e., 1 April–30 September), daily minimum temperatures range from −15.5–24.0°C (mean = 9.8°C, *SD* = 6.4°C), daily maximum temperatures range from −2.0–39.5°C (mean = 22.4°C, *SD* = 6.6°C), and daily precipitation ranges from 0–85.0 mm (mean = 3.4 mm, *SD* = 7.2 mm; Thornton et al., 2018). During the course of this study, daily minimum and maximum temperatures ranged from −11.0 to 20.5°C (mean = 9.7°C, *SD* = 6.1°C) and −1.0–34.5°C (mean = 22.1°C, *SD* = 6.5°C), respectively; daily precipitation ranged from 0–58.0 mm (mean = 3.3 mm, *SD* = 7.5 mm; Thornton et al., 2018). Data used in this study were collected at the Buena Vista, Paul Olson, and Leola sites. More detailed descriptions of the study area, history, population status, and management of prairie chickens in Wisconsin can be found in Niemuth (2000), Hull et al. (2011), Broadway (2015), and Hardy et al. (2018).

### 2.2 Land cover and management data

We obtained spatially explicit land cover data for the entire CWGCA and management records for the three properties where demographic data were collected (Buena Vista, Paul Olson, and Leola). To characterize land cover in the CWGCA, we used the United States Department of Agriculture (USDA) National Agricultural Statistics Service's Cropland Data Layers (CDL) from 2007–2009 and 2014–2015 (USDA National Agricultural Statistics Service, 2015). The CWGCA contained 88 unique land cover types during this period; we considered development and open water as unavailable to prairie chickens and reclassified remaining land cover data into four simplified classes based on structural characteristics (Appendix S1: Table S1): cultivated row crops, open “grassy” habitats (e.g., remnant prairies, pastures, hay/small grain fields), trees/shrubs (including forest, shrubland, and various cultivated tree crops) and herbaceous wetlands (i.e., areas dominated by perennial herbaceous vegetation where the soil or substrate is periodically saturated or covered with water, including wet prairies and sedge meadows). Because cranberries were classified poorly by CDL, we manually digitized cranberry bogs from the USDA’s National Agriculture Imagery Program (NAIP) imagery. As NAIP imagery was not available for every year, we used the 2008, 2013, and 2015 imagery to characterize cranberries during 2007–2009, 2014, and 2015, respectively.

We obtained habitat management records consisting of either ArcGIS shapefiles or hard‐copy maps depicting locations of all management actions conducted at a site during each year from property managers, digitized if necessary, and converted to raster format (30‐m cell size); these records covered the time period from 1981–2015 (Buena Vista and Leola: 1981–2015, Paul Olson: 2006–2010 and 2013–2015). We considered eight management practices ranging from targeted removal of woody vegetation (mechanical brush/tree removal and herbicide spraying) to maintenance of disturbance processes in grassland systems (prescribed burning from mid‐March through mid‐May, mowing, and rotational grazing with light‐to‐moderate stocking rates) to practices associated with agricultural land use (hay cutting from mid‐July through mid‐August, sharecropping, and plowing/disking treatments with the field subsequently left idle; hereafter plow/disk/idle).

### 2.3 Hen monitoring

In total, 237 prairie chicken hens were monitored by the Wisconsin Department of Natural Resources and University of Wisconsin‐Stevens Point during the nesting and brood‐rearing seasons of 2007–2009 and 2014–2015. During March‐July 2006–2009, 110 hens were captured on or near leks in Minnesota, USA, using either baited walk‐in funnel traps or drop nets and fitted with unique serial‐numbered metal leg bands and a 12‐g necklace style radio transmitter with an 8‐hr mortality switch (Model TS‐25, Telemetry Solutions, Inc.). Hens were then relocated during summer molt in August‐September using radio telemetry, recaptured using spotlights and long‐handled nets, and translocated to Buena Vista (Hull et al., 2013). An additional 65 hens were captured at Buena Vista following the same protocol and released at the capture site. During 2007–2009, 15 hens (10 from Minnesota and 5 from Wisconsin) either dispersed out of detection range or had failed transmitters; these individuals were excluded from analyses. Similarly, 33 hens that died during the first overwintering period (1 October–30 March) following their initial release were also excluded from analysis. During March‐May of 2014 and 2015, 62 hens were captured at Buena Vista (*N* = 42) and Paul Olson (*N* = 20) using walk‐in style traps (Schroeder & Braun, 1991), fitted with unique leg bands and a 16‐g necklace style transmitter with a 6‐hr mortality switch (Model #A3960, Advanced Telemetry Systems, Inc.), and released at the capture location (Broadway, 2015). Captured hens were aged, and sex was confirmed based on plumage characteristics (Johnson et al., 2011). During both periods, radio‐marked hens were tracked ≥3 times per week from 1 April–30 September with portable receivers (Model R2000, Advanced Telemetry Systems) and 3‐element folding Yagi antennas, and their locations were estimated using maximum‐likelihood triangulation methods (Lenth, 1981). Hen locations with error polygons ≤16.19 ha (i.e., a quarter‐quarter section) were included in the study.

### 2.4 Nest monitoring

In all years, nests were located by homing in on hens for which we had three consecutive equivalent locations. A total of 192 prairie chicken nests were monitored during 2007–2009 and 2014–2015 at Buena Vista (*N* = 155), Paul Olson (*N* = 26), and Leola (*N* = 11); 18.2% of nests (35/192) were renesting attempts after an initial nest had failed. Hens were flushed from nests once during early incubation to determine clutch size and estimate incubation stage by either floating or candling the eggs (Hess et al., 2012; McNew et al., 2009). Expected hatch dates were estimated assuming an average clutch size of 12 eggs (McNew et al., 2012) and average incubation period of 24 days (Johnson et al., 2011). During 2014–2015, observers placed a Thermochron iButton data‐logger (Model DS 1921G) in the bowl of each nest and within 100 m of each nest to simultaneously record ambient temperatures, allowing for more precise estimation of hatch or fail dates (Hartman & Oring, 2006). Observers continued to monitor incubating hens from a distance via radio telemetry until the nest either hatched or failed. Frequency of monitoring increased as the estimated hatch date approached. If a hen was located away from her nest site on two consecutive occasions, observers approached the nest to determine reproductive status (i.e., ≥1 egg hatched or nest failure) and, if possible, cause of nest failure. When available, we subsequently examined iButton data to determine the exact date of hatch or failure.

### 2.5 Brood monitoring

During 2014–2015, we monitored 23 prairie chicken broods representing a total of 222 chicks at Buena Vista (*N* = 14), Paul Olson (*N* = 5), and Leola (*N* = 4). Hens tending broods were located within 30 min of local sunrise at regular weekly intervals to assess brood status (Goddard & Dawson, 2009). During these weekly encounters, observers counted as many chicks as possible immediately after flushing the attending hen and recorded the flush location using a handheld GPS unit. For each brood, weekly flush counts were conducted when the chicks were 7 days of age and continued until chicks reached 70 days of age or the brood was lost. Broods were considered lost if no chicks were sighted during two consecutive weekly flush counts and hen behavior suggested that she was no longer tending chicks (e.g., flocking behavior). In cases where hen behavior was inconclusive, a third flush count was conducted to confirm that the brood was lost.

### 2.6 Home ranges and nest buffers

For each hen, we defined annual breeding season home ranges as the minimum convex polygon (MCP) bounding all known locations from 1 April–30 September during each year. For brood home ranges, we included the nest location, flush count locations, and all locations of the brood hen spanning the period from the brood's hatch date to 70 days from hatch, or, in the case of failed broods, the date that the brood was determined to have failed. For purposes of generating home ranges, we included all hens and broods that had at least 3 distinct locations. Although previous studies of prairie chicken nest survival have often focused on small‐scale or microsite characteristics, we note that prairie grouse nest success can be influenced by landscape composition at spatial scales up to 1600m from the nest (Manzer & Hannon, 2005). Consequently, we characterized the landscape surrounding nests by buffering each nest location with an 1182‐m radius circular buffer, corresponding to the average area of a hen's breeding season home range (439 ha). For purposes of calculating hen home‐range size, we only considered hens with at least 30 known locations that survived the entire breeding season (*N* = 92). We chose these criteria because (a) including hens that die early in the season could bias estimates of home‐range size low, and (b) there is a positive relationship between number of tracking locations and prairie chicken home‐range size until ≈30 locations have accrued (Patten et al., 2011).

### 2.7 Resource selection function analyses

We used exponential resource selection functions (RSF; Manly et al., 2002) to investigate habitat selection behavior. We aggregated Buena Vista and Leola into a single landscape because these two wildlife areas are <5 km from each other and our radio‐tracking data indicated that hens occasionally moved between Buena Vista and Leola. Paul Olson remained a separate landscape because it is isolated from the more southerly management areas by the Wisconsin River and metropolitan area of Stevens Point and Wisconsin Rapids. We delineated these two landscapes based on the minimum convex polygons that included all known hen locations recorded during 2007–2015 at Buena Vista/Leola and Paul Olson, respectively. We extracted values from our land cover and management rasters (30 m‐cell) to points located at the center of each raster cell that fell within the boundaries of either landscape (235,597 and 159,002 available points at Buena Vista/Leola and Paul Olson, respectively).

We modeled habitat selection by hens at the landscape scale, nest‐site selection within hen home ranges, and selection for brood‐rearing areas within hen home ranges. The first analysis corresponds to 2nd‐order selection, whereas the latter two analyses represent 3rd‐order selection (Johnson, 1980). For hen habitat selection, we randomly sampled without replacement 100 available points per used point from the corresponding landscape. For nest models, we compared characteristics at the nest location to all available points within the corresponding hen's home range. For brood models, we compared known locations of the brood to all available points that fell within the brood hen's home range. For all analyses, we included a random intercept term to account for differences in the number of used locations among hens (Gillies et al., 2006).

We modeled home range, brood‐rearing, and nest‐site selection using a multistage model selection approach that incorporates elements of the build‐up and secondary subsets approaches recommended by Morin et al. (2020). For each of the three life‐history stages (hen, nest, and brood), we first examined land cover and management variables in separate subsets and ranked models based on Akaike's information criterion corrected for small sample size (AIC*_c_*; Burnham & Anderson, 2002). Within each subset, we considered variables with ∆AIC*_c_* ≤ 2 units of the top‐ranked to be strongly supported and subsequently used the build‐up approach to develop additional models considering additive effects of these variables. We then combined the best‐supported model structures from each subset into a final model set to simultaneously consider land cover and management. For each of the three selection analyses, the land cover subset initially included seven candidate models: a model that included a categorical variable for each land cover class (treating grassland as the reference category), separate models considering each of the five land cover classes individually (treating all other classes combined as the reference category), and the null (intercept‐only) model. The management subset initially included a model for each of the eight management practices in the current year (*t*) and during the previous year (*t* − 1) plus the null model, for a total of 17 models per life‐history stage. In a few cases, complete separation occurred; we excluded these models from further consideration. All RSF models were fit using the glmmTMB package in R version 4.0.1 (Brooks et al., 2017; R Foundation for Statistical Computing, 2020). Results are presented as odds ratios ± *SE* and associated 95% confidence intervals.

### 2.8 Survival analyses

We modeled nest, hen, and brood survival probabilities using Program MARK (White & Burnham, 1999). We used the nest survival model (Dinsmore et al., 2002) to estimate daily survival rates of nests and hens, and the young survival model (Lukacs et al., 2004) to estimate weekly survival rates of broods. For hens, we extrapolated the daily survival rate to the entire nesting and brood‐rearing season (1 April–30 September, 183 days) and used the delta method to obtain estimates of variance for the extrapolated survival estimates (Powell, 2007). To evaluate the influence of land cover and habitat management on vital rates, we used Fragstats version 4.2.1 (McGarigal et al., 2012) to calculate the proportion of each home range and nest buffer that was composed of each management practice or cover type in each year.

As above, we modeled relationships between vital rates and land cover/management practices using a multistage model selection approach based on AIC*_c_* as described above. For broods, we first evaluated the effects of year and brood age in weeks (linear or quadratic) on detection probability (*p*) assuming constant survival (*φ*) prior to developing any survival models. We then used the best‐supported model structure for detection probability in all subsequent brood models. We initially considered three subsets for each vital rate: (a) a subset considering the effects of year, site, hen age (i.e., second‐year or after‐second‐year), hen origin (i.e., native Wisconsin hens or translocated hens from Minnesota), brood age, and/or first nest versus. renest, as appropriate; (b) linear and quadratic relationships with each land cover class; and (c) each management practice conducted in the current and previous year. As above, we used the build‐up approach within each subset to examine additive effects of strongly supported variables and combined the best‐supported model structures from each subset into a final model set for each vital rate. To avoid multicollinearity, we did not include highly correlated variables (i.e., |*r*| > 0.7; Dormann et al., 2013) in the same model. Results are presented as regression coefficient estimates (*β*) ± *SE* and associated 95% confidence intervals.

## 3 RESULTS

A total of 8,414 observations of 189 hens were included in the study; 614 of these locations represented hens tending broods. Several hens were monitored in multiple years: Sample sizes for Buena Vista/Leola in 2007–2009 and 2014–2015 were 58, 43, 35, 19, and 28 hens, respectively; sample sizes for Paul Olson in 2014–2015 were 8 and 14 hens, respectively. The number of locations per hen ranged from 3 to 218 (mean = 51.30, *SD* = 41.92), and the number of locations per brood ranged from 4 to 79 (mean = 26.70, *SD* = 23.76). Error polygons for telemetry locations ranged from 0 to 16.19 ha in size (mean = 0.48, *SD* = 1.06). Observers recorded 70 hen mortalities and 118 nest failures; 6 of 23 broods fledged at least one chick. Landscape composition varied substantially among home ranges and nest buffers, with each land cover class representing 0%–100% of total cover within hen home ranges, 0%–94.08% within brood home ranges, and 0%–94.30% within nest buffers (Table 1). The prevalence of management practices was also highly variable, with each practice representing 0%–87.09% of total cover for hen home ranges, 0%–52.09% for brood home ranges, and 0%–34.18% for nest buffers (Table 1).

**Table 1:** Prevalence of land cover types and management practices in greater prairie chicken home ranges and 1,182 m‐radius nest buffers in the Central Wisconsin Grassland Conservation Area, Wisconsin, USA, 2007–2015

	Hen home ranges		Brood home ranges		Nest buffers	
	Range	Mean (*SD*)	Range	Mean (*SD*)	Range	Mean (*SD*)
Land cover						
Cranberries	0–18.91	0.84 (2.78)	0–0.1	0 (0.02)	0–15.53	1.18 (2.72)
Grassland	12.28–100	70.31 (21.30)	17.50–92.08	61.33 (20.72)	6.83–94.30	60.13 (21.14)
Herbaceous wetlands	0–21.84	3.61 (3.58)	0–33.75	6.77 (8.02)	0.12–16.43	3.98 (3.23)
Row crops	0–73.51	12.28 (13.06)	0–41.66	14.77 (12.57)	0.08–64.08	15.42 (14.76)
Trees/shrubs	0–64.10	7.86 (9.64)	0–57.30	13.83 (14.69)	0.16–50.54	15.42 (14.76)
Management practice						
Brush/tree removal	0–42.27	2.89 (6.12)	0–2.30	0.10 (0.48)	0–15.89	2.21 (4.11)
Grazing	0–46.66	2.82 (7.84)	0–9.34	1.02 (2.43)	0–34.18	2.47 (5.57)
Hay cutting	0–52.38	0.50 (3.76)	–	–	0–3.80	0.31 (0.85)
Herbicide spraying	0–87.09	3.43 (9.47)	0–19.77	2.36 (5.26)	0–27.86	2.62 (4.13)
Mowing	0–45.61	1.16 (4.68)	0–52.09	2.67 (10.86)	0–13.53	1.18 (3.10)
Plow/disk/idle	0–47.90	0.44 (3.90)	–	–	0–12.06	0.21 (1.21)
Prescribed burning	0–22.48	0.87 (3.37)	0–25.75	1.48 (5.57)	0–14.67	0.66 (2.58)
Sharecropping	0–6.46	0.21 (0.73)	–	–	0–4.55	0.32 (0.82)

### 3.1 Hen home‐range selection

**Figure 3:**
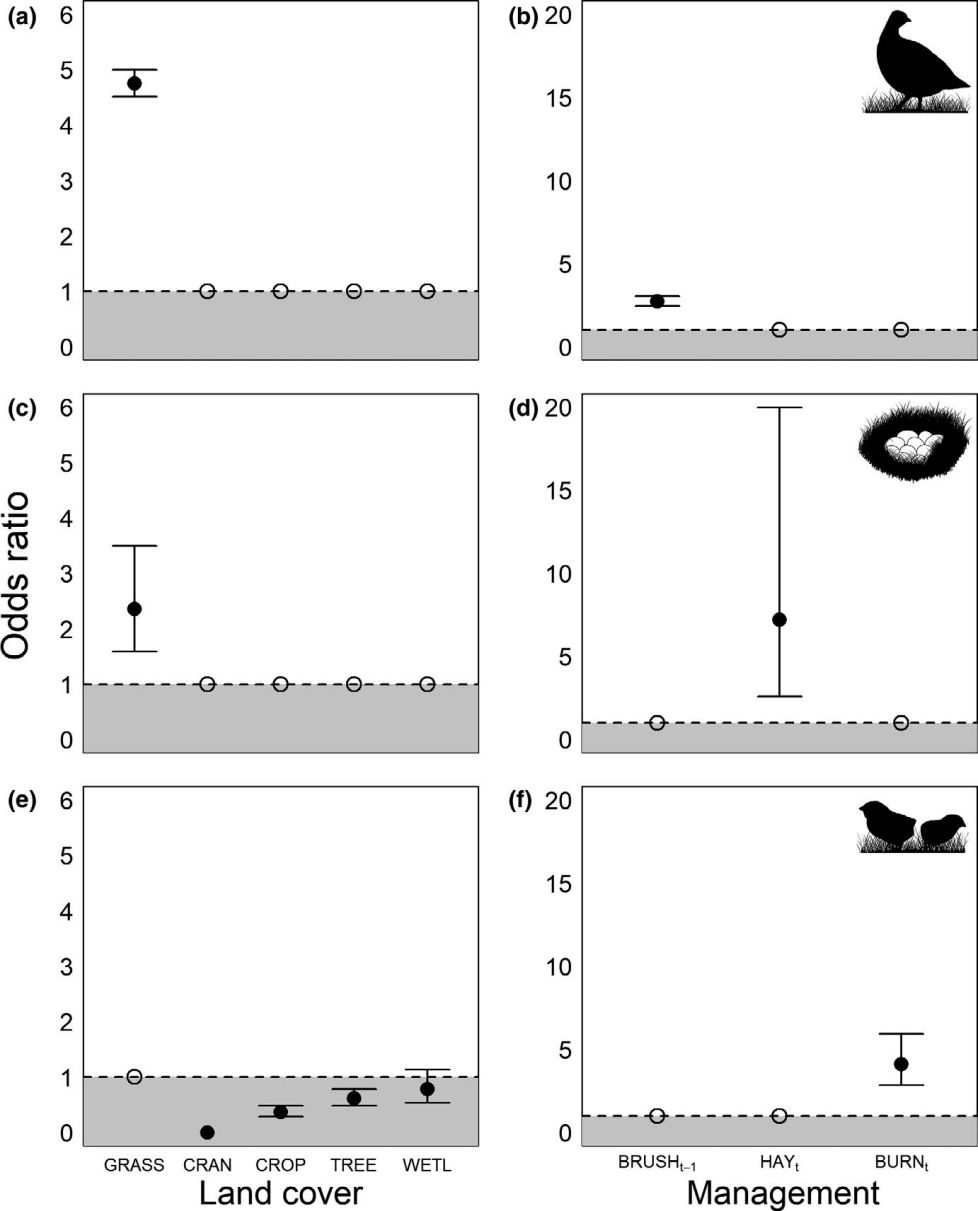
Odds ratios for the effects of land cover type (GRASS = grasslands, CRAN = cranberry bogs, CROP = row crops, TREE = trees/shrubs, WETL = herbaceous wetlands) and management practices (BRUSH = brush/tree removal, HAY = hay cutting, BURN = prescribed burning) on habitat selection for greater prairie chicken hens (a, b), nest sites (c, d), and broods (e, f) at three sites in the Central Wisconsin Grassland Conservation Area, Wisconsin, USA, 2007–2015. Open circles (○) represent a "baseline" probability of use = 1 (dashed line), values ≥ 1 represent selection, values ≤ 1 represent avoidance (gray shaded area), and whiskers denote 95% confidence intervals. Although prairie chickens were more likely to use grassland relative to other cover types during all life‐history stages, the importance of management practices varied among life‐history stages

During the first round of home‐range selection modeling, the model representing selection of grassland over all other land cover classes combined received overwhelming support in the land cover subset (*w_i_* = 1.0; Appendix S2: Table S1). Considering management actions, hens selected for areas where brush and trees had been removed during the previous year, and this was the only model with substantial support (*w_i_* = 1.0; Appendix S2: Table S1). The model including grassland cover and brush/tree removal during the previous year received the most support in our final model set (*w_i_* = 1.0, ∆AIC*_c_* ≥ 245.73; Table 2). Hens were 4.75 ± 1.03 (95% CI = 4.51–5.00) times more likely to select grassland compared to other land cover types (Figure 3a) and 2.71 ± 1.06 (95% CI = 2.43–3.03) times more likely to occupy areas where brush and trees had been removed during the previous year (Figure 3b).

**Table 2:** Final model rankings considering the effects of land cover and management practices on greater prairie chicken habitat selection at three sites in the Central Wisconsin Grassland Conservation Area, Wisconsin, USA, 2007–2015

Model^a^	AIC*_c_*	∆AIC*_c_*	*w_i_*	Likelihood	*K*	Deviance
Hen home‐range selection						
GRASS+BRUSH*	89,657.3	0	1	1	4	89,649.3
GRASS	89,903.1	245.73	0	0	3	89,897.1
BRUSH*	93,937.8	4,280.5	0	0	3	93,931.8
NULL MODEL	94,411.7	4,754.32	0	0	2	94,407.7
Nest‐site selection						
GRASS+HAY	3,361.93	0	0.69	1	4	3,353.93
CRAN+CROP+TREE+WETL +HAY	3,363.77	1.84	0.28	0.4	7	3,349.77
GRASS	3,368.58	6.64	0.02	0.04	3	3,362.58
CRAN+CROP+TREE+WETL	3,370.36	8.43	0.01	0.01	6	3,358.36
HAY	3,381.07	19.14	0	0	3	3,375.07
NULL MODEL	3,388.5	19.96	0	0	2	3,384.54
Brood‐rearing site selection						
CRAN+CROP+TREE+WETL +BURN	6,499.48	0	1	1	7	6,485.48
CRAN+CROP+TREE+WETL	6,543.08	43.6	0	0	6	6,531.08
BURN	6,567.78	68.3	0	0	3	6,561.78
NULL MODEL	6,612.05	112.57	0	0	2	6,608.05

Note

Models were ranked based on Akaike's information criterion corrected for small sample size (AIC*_c_*). Akaike weights (*w_i_*), relative model likelihoods, number of estimated parameters (*K*), and model deviance values are presented. Asterisks (*) denote management practices that occurred during the previous year.

^a^ Cranberry bogs (CRAN), row crops (CROP), open grassland habitats (GRASS), trees/shrubs (TREE), herbaceous wetlands (WETL), brush/tree removal (BRUSH), prescribed burning (BURN), hay cutting (HAY).

### 3.2 Nest‐site selection

For nest‐site selection, the land cover model including only grass cover received the most support (*w_i_* = 0.70), but the model including all land cover types was also competitive (*w_i_* = 0.29, ∆AIC*_c_* = 1.79; Appendix S2: Table S2). The management model representing hay cutting during the current year received the most support (*w_i_* = 0.69, ∆AIC*_c_* ≥ 4.03; Appendix S2: Table S2). The best‐supported model in our final set included grass cover and hay cutting (*w_i_* = 0.69), but the model including all land cover types and hay cutting was also competitive (*w_i_* = 0.28, ∆AIC*_c_* = 1.84; Table 2); we present results from the more parsimonious model here. Hens were 2.36 ± 1.22 (95% CI = 1.59–3.49) times more likely to nest in grass cover compared to other cover types (Figure 3c) and 7.19 ± 1.68 (95% CI = 2.59–19.96) times more likely to nest in hay fields (Figure 3d).

### 3.3 Brood‐rearing site selection

During the initial model selection for brood‐rearing sites, the model including all land cover classes received strong support (*w_i_* = 0.99; Appendix S2: Table S3). Out of all management actions, prescribed burning during the current year was the best‐supported model (*w_i_* = 0.99; Appendix S2: Table S3). The top model in our final set, which included all land cover classes plus prescribed fire, received overwhelming support (*w_i_* = 1.0, ∆AIC*_c_* ≥ 43.60; Table 2). When rearing broods, hens avoided locations in cranberries, row crops, trees/shrubs, and herbaceous wetlands (odds ratios = 2.69 × 10^–7^, 0.37, 0.61, and 0.78, respectively; Figure 3e). Hens were 4.10 ± 0.1.21 (95% CI = 2.84–5.94) times more likely to brood chicks in areas that were burned during the current year (Figure 3f).

### 3.4 Hen survival

Models considering hen age, hen origin, and the combination of hen age and origin were competitive in our first subset of hen survival models (Appendix S3: Table S1). Two models (quadratic relationships with grass cover and row crops) were competitive in the land cover subset; we did not combine these variables as they were highly correlated (*r* = −0.79), but we retained both models for additional analyses. Three management practices (brush/tree removal during the current and previous years, and prescribed burning during the current year) were competitive, and three model structures representing combinations of these variables were retained for final analyses (Appendix S3: Tables S1, S4).

**Figure 4:**
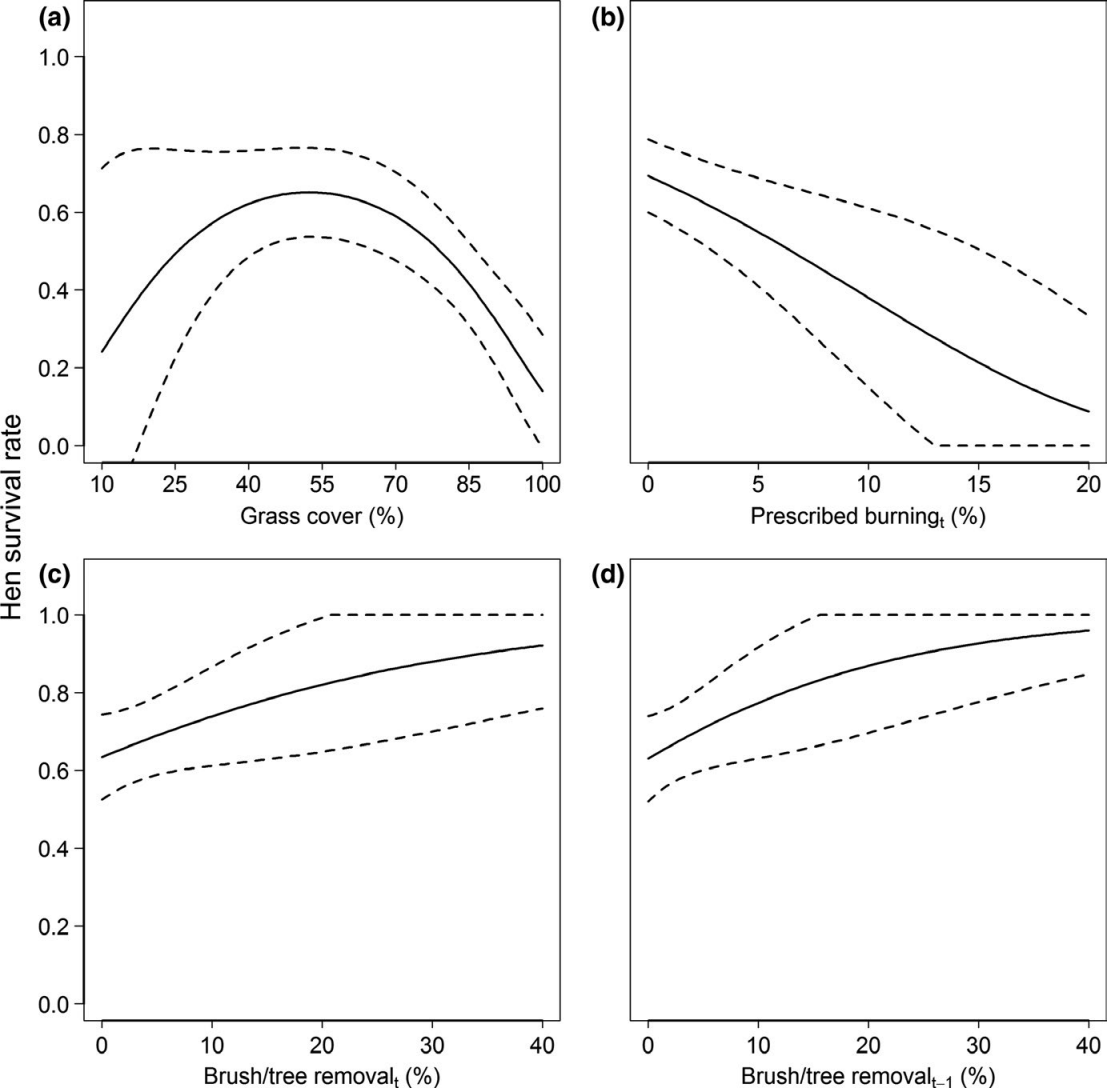
Effects of grassland cover (a), prescribed burning during the current year (b), and brush/tree removal in the current (c) and previous (d) year on the breeding season survival (1 April‐30 September) of greater prairie chicken hens in the Central Wisconsin Grassland Conservation Area, Wisconsin, USA, 2007–2015. Solid lines denote mean values, and dashed lines denote 95% confidence intervals

There was some uncertainty in our final model set, with three competitive models (∆AIC*_c_* = 1.65–1.88; Table 3). The best‐supported model received ≈22% of the total model weights and included the quadratic effect of grass cover, brush/tree removal during the current and previous years, and prescribed burning during the current year. The second‐ and third‐ranked models included the same land cover and management variables, but also included weak effects of hen origin (*β_OriginMN_* = 0.16 ± 0.28, 95% CI = −0.39–0.72) and hen age (*β_AgeSY_* = −0.11 ± 0.33, 95% CI = −0.76 to 0.53), respectively. Weights for these models ranged from 0.09–0.10. The best‐supported model suggested that hen survival varied according to a quadratic relationship with grass cover (*β_GRASS_* = −0.42 ± 0.14, 95% CI = −0.69 to −0.14; *β_GRASS_^2^* = −0.31 ± 0.11, 95% CI = −0.53 to −0.09), with highest survival at ≈ 55% cover. Hen survival was positively associated with brush/tree removal (*β_BRUSH(t)_* = 0.33 ± 0.17, 95% CI = −0.01–0.67; *β_BRUSH(t‐1)_* = 0.40 ± 0.21, 95% CI = −0.01 to 0.81) and negatively associated with prescribed fire (*β_BURN(t)_* = −0.25 ± 0.10; 95% CI = −0.44 to −0.05). Coefficient estimates from the other competitive models were similar; relationships from the top model are presented in Figure 4. Assuming mean values of all covariates, we estimated hen breeding season survival to be 0.68 ± 0.05 (95% CI = 0.58–0.77).

**Table 3:** Final rankings for competitive models (∆AIC*_c_* ≤ 2) considering the effects of land cover and management practices on greater prairie chicken survival rates at three sites in the Central Wisconsin Grassland Conservation Area, Wisconsin, USA, 2007–2015

Model^a^	AIC*_c_*	∆AIC*_c_*	*w_i_*	Likelihood	*K*	Deviance
Hen survival						
GRASS+GRASS^2^+BRUSH+BRUSH*+BURN	810.54	0.00	0.22	1.00	6	798.54
HEN.ORIGIN+GRASS+GRASS^2^+BRUSH +BRUSH*+BURN	812.19	1.65	0.10	0.44	7	798.18
HEN.AGE+GRASS+GRASS^2^+BRUSH +BRUSH*+BURN	812.42	1.88	0.09	0.39	7	798.42
NULL MODEL	824.80	14.26	0.00	0.00	1	822.79
Nest survival						
YEAR+CROP+TREE	918.98	0.00	0.05	1.00	7	904.95
YEAR+GRASS +TREE	919.22	0.24	0.04	0.89	7	905.18
YEAR+RENEST +CROP+TREE	920.09	1.11	0.03	0.57	8	904.05
YEAR+TREE	920.20	1.21	0.02	0.54	6	908.17
YEAR+RENEST +GRASS+TREE	920.52	1.54	0.02	0.46	8	904.48
YEAR+GRASS	920.55	1.56	0.02	0.46	6	908.52
YEAR++CROP +TREE+PDI.L	920.75	1.77	0.02	0.41	8	904.71
YEAR+CROP +TREE+BRUSH.L	920.92	1.94	0.02	0.38	8	904.88
NULL MODEL	925.89	6.91	0.00	0.03	1	923.89
Brood survival						
φ(SITE+TREE+TREE^2^+GRAZE)p(YEAR)	470.20	0.00	0.80	1.00	8	452.68
NULL MODEL	567.65	97.46	0.00	0.00	2	563.53

Note

Models were ranked based on Akaike's information criterion corrected for small sample size (AIC*_c_*). Akaike weights (*w_i_*), relative model likelihoods, number of estimated parameters (*K*), and model deviance values are presented. Asterisks (*) denote management practices that occurred during the previous year. Rankings for all models are presented in Appendix S3: Tables S4–S6.

^a^ Site (SITE), year (YEAR), hen age (HEN.AGE), hen origin (HEN.ORIGIN), row crops (CROP), grassland (GRASS), trees/shrubs (TREE), mechanical brush/tree removal (BRUSH), prescribed burning (BURN), grazing (GRAZE), plow/disk/idle (PDI).

### 3.5 Nest survival

In our first nest survival model subset, model structures including annual variation and/or an effect of first nest versus renest were competitive (Appendix S3: Table S2). Nest survival declined appreciably over the course of the study: The average daily survival rate of nests fell from 0.9649 ± 6.6734 × 10^–3^ in 2007 to 0.9313 ± 1.4734 × 10^–2^ in 2015. There was substantial model selection uncertainty in the land cover subset, and model structures containing various combinations of cranberries, crops, grassland, trees, and wetlands were all retained for the final model set (Appendix S3: Table S2). Similarly, several model structures that included combinations of brush/tree removal and plow/disk/idle treatments during the current and previous years were competitive and carried forward to the final analysis (Appendix S3: Table S2).

**Figure 5:**
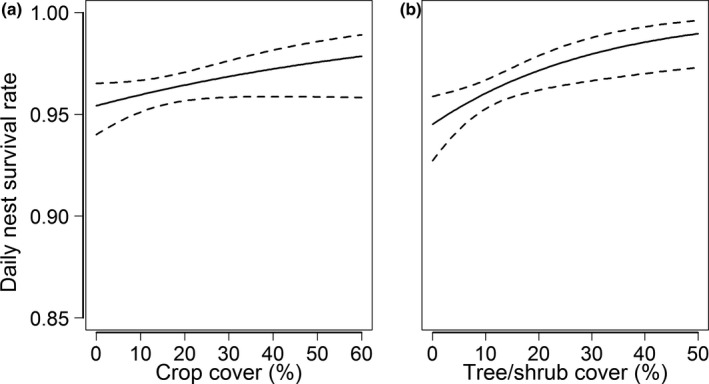
Effects of row crops (a) and tree/shrub cover (b) on the daily survival rate of greater prairie chicken nests in the Central Wisconsin Grassland Conservation Area, Wisconsin, USA, 2007–2015. Estimates for 2007 are shown. Solid lines denote mean values, and dashed lines denote 95% confidence intervals

There was considerable uncertainty in our final model set, with eight competitive models collectively representing 0.21 of the total model weights (Table 3, Appendix S3: Table S4). All eight competitive models contained a year effect, seven models included tree cover, four models included crops, three models included grassland, two models included renesting, and plow/disk/idle and brush/tree removal during the previous year were each included in one model. The highest‐ranked model included a year effect, a weak positive relationship with row crops (*β_CROP_* = 0.19 ± 0.11, 95% CI = −0.02–0.41; Figure 5a), and a positive relationship with tree/shrub cover (*β_TREE_* = 0.39 ± 0.14, 95% CI = 0.12–0.67; Figure 5b). Coefficient estimates for year, row crops, and trees/shrubs were similar among the competitive models. Coefficients for grassland and renesting were negative in all models, ranging from −0.32 ± 0.12 (95% CI = −0.56 to −0.08) to −0.21 ± 0.13 (95% CI = −0.46 to 0.05) and −0.22 ± 0.24 (95% CI = −0.70 to 0.24) to −0.20 ± 0.24 (95% CI = −0.67 to 0.26), respectively. Brush/tree removal and plow/disk/idle treatments during the previous year both had extremely weak relationships with nest survival despite appearing in competitive models (*β_BRUSH(t‐1)_* = 0.03 ± 0.13, 95% CI = −0.21 to 0.28; *β_PDI(t‐1)_* = 0.06 ± 0.14, 95% CI = −0.20 to 0.33).

### 3.6 Brood survival

**Figure 6:**
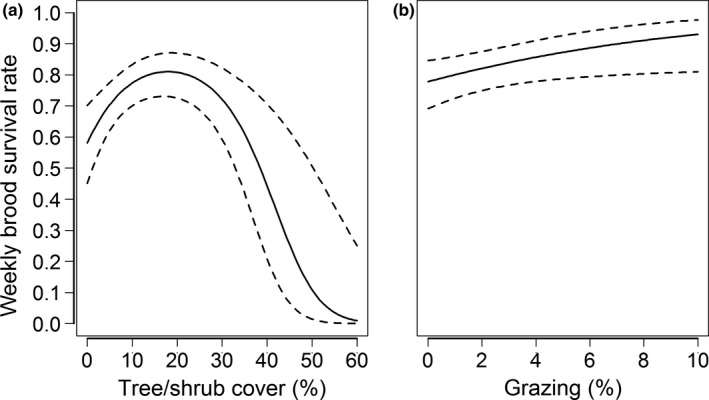
Effects of tree/shrub cover (a) and rotational grazing (b) on the weekly survival rate of greater prairie chicken broods in the Central Wisconsin Grassland Conservation Area, Wisconsin, USA, 2007–2015. Estimates for Buena Vista are shown. Solid lines denote mean values, and dashed lines denote 95% confidence intervals

The model accounting for annual differences in detection probability received strong support (*w_i_* = 1.0; Appendix S3: Table S3); we therefore included a “year” effect on *p* in subsequent models. We found strong evidence for differences in survival among sites (*w_i_* = 1.0; Appendix S3: Table S3). A quadratic relationship with tree/shrub cover was the only variable to receive appreciable support in the candidate model subset for land cover (*w_i_* = 1.0) and grazing during the current year was the only variable to receive appreciable support in the candidate model subset for management (*w_i_* = 1.0; Appendix S3: Table S3). When we combined land cover and management variables in our final model set, the highest‐ranked model included tree/shrub cover and grazing and received strong support (*w_i_* = 0.80, (∆AIC*_c_* ≥ 2.77; Appendix S3: Table S6). The final model suggested brood survival was greatest at ≈15%–20% tree/shrub cover (*β_TREE_* = 0.42 ± 0.20, 95% CI = 0.04–0.81; *β_TREE_^2^* = −0.75 ± 0.21, 95% CI = −1.17 to −0.33; Figure 6a) and was positively influenced by grazing (*β_GRAZE(t)_* = 0.32 ± 0.15, 95% CI = 0.03–0.62; Figure 6b). Detection probability during flush counts was comparable in 2014 (0.71 ± 0.05, 95% CI = 0.59–0.81) and 2015 (0.73 ± 0.06, 95% CI = 0.61–0.83), and brood survival was considerably lower at Leola compared to Buena Vista or Paul Olson. Assuming average values of tree/shrub cover and grazing, weekly survival rates for broods at Leola, Buena Vista, and Paul Olson were 0.1577 ± 0.0831, 0.8844 ± 0.0235, and 0.8852 ± 0.0269, respectively. Thus, the corresponding probabilities of a chick surviving to 70 days of age were 9.53 × 10^–9^ ± 5.02 × 10^–8^ (95% CI = 0–1.08 × 10^–7^), 0.29 ± 0.08 (95% CI = 0.14–0.45), and 0.30 ± 0.09 (95% CI = 0.12–0.47) for Leola, Buena Vista, and Paul Olson.

## 4 DISCUSSION

In this study, we estimated relationships among land use, grassland management practices, habitat selection, and survival rates for remnant populations of greater prairie chickens in central Wisconsin, USA. Although responses to land cover and management were complex, we identified four key results that have important implications for prairie chicken management. First, prairie chickens selected different management practices during different life‐history stages. Second, hen, nest, and brood survival rates were each influenced by different management practices. Third, only a single management practice was associated with both habitat selection and survival during the same life‐history stage. Finally, we identified one management practice that was selected during one life‐history stage influenced survival during a different life‐history stage. Collectively, our results suggest that failure to consider multiple population responses may lead to an incomplete understanding of the effects of management efforts, or, at worst, the creation of ecological traps (Hale & Swearer, 2017).

Although prairie chickens selected grassland habitat over other cover types during all life‐history stages, none of the management practices we examined had a consistent influence on habitat selection or survival during more than one life‐history stage. Hens, for example, selected recently burned areas for rearing broods, but neither home‐range selection nor nest‐site selection were strongly influenced by prescribed fire. Similarly, hens showed no general affinity for hay fields at the landscape scale, but demonstrated strong selection for hay fields when selecting nest sites within their home range, likely cueing in on tall dense vegetation (McNew et al., 2014, 2015) that can bolster nest survival rates (Hovick et al., 2015). The amount of brush/tree removal (in both the current and previous year) within hen home ranges had a positive influence on hen survival, but did not appear to influence brood survival and had only a weak and somewhat ambiguous relationship with nest survival.

Likewise, grazing appeared to bolster brood survival rates, but had no appreciable influence on hen or nest survival. Prairie chicken brood‐rearing habitat is often associated with disturbance such as haying, mowing, or grazing (Svedarsky, 1988), which facilitates movement (Johnson et al., 2011) and foraging (Jones, 1963) by precocial chicks at or near ground level. Collectively, these results underscore the hierarchical nature of habitat selection behavior (Johnson, 1980), and we conclude that it may be necessary to manage areas using several different practices to provide prairie chicken habitat during multiple life‐history stages, broadly supporting previous research highlighting the importance of landscape heterogeneity for grassland bird management (Fuhlendorf et al., 2006; Rahmig et al., 2009; Sandercock et al., 2015).

Ecological theory predicts that, when possible, animals should select habitats that promote high survival or reproductive success (Fretwell & Lucas, 1969). However, we found that within each life‐history stage, only one management practice and only a limited number of land cover classes were associated with both habitat selection and survival, hinting at a possible decoupling of habitat selection and habitat quality during one or more life‐history stages. At the landscape scale, hens actively selected grassland, and particularly areas where brush and trees had been removed during the previous year, which tended to increase survival. However, it is worth noting that individuals with ≈ 55% grass cover within their home ranges had the greatest survival rates, suggesting that some amount of nongrassland habitat might be beneficial. For example, prairie chickens occasionally use herbaceous wetlands (e.g., wet prairies or sedge meadows; Wisconsin Department of Natural Resources, 2015), and, to a lesser extent, shrubs, as daytime loafing and night roosting locations (Hamerstrom et al., 1957; Toepfer & Eng, 1988), possibly because they offer favorable thermal microclimates, additional sources of food, or concealment from predators, which may in turn increase survival. Additionally, hens consistently selected nest sites in grassland habitat, but we detected a negative relationship between grass cover in the surrounding landscape and nest survival; instead, nest survival was positively associated with both row crop and tree/shrub cover. These results are quite surprising and do not align well with previous research or with prairie grouse biology in general. It is possible that grasslands in agricultural landscapes, although attractive, may in fact be sink habitats due to novel conditions associated with extensive human modification. In extreme cases, such grasslands might act as ecological traps (Battin, 2004; Hale & Swearer, 2017). On the other hand, it may simply be the case that we did not identify the correct scale of effect (i.e., the spatial extent at which landscape structure best predicts population response; Jackson & Fahrig, 2012) for nest survival in this system. Further investigation of this topic that includes multiple spatial scales (Jackson & Fahrig, 2015) is certainly justified and may help provide better context for these results.

Finally, hens selected recently burned areas when rearing broods, but prescribed fire within hen home ranges tended to depress hen survival and offered no apparent advantage in terms of brood survival. Although prairie grouse populations are generally most sensitive to changes in nest and brood survival (Hamerstrom et al., 1957; Wisdom & Mills, 1997), we note that some declining populations of prairie chickens may actually be more sensitive to changes in adult survival (McNew et al., 2012, M.A. Hardy, *unpublished data*). Consequently, management practices focused on prairie chicken broods may have unintended detrimental effects by depressing adult survival rates. It is therefore critical that managers consider different patterns in habitat selection and their demographic consequences across multiple life‐history stages within the context of the population targeted for management before engaging in habitat manipulations.

Our estimates of hen, nest, and brood survival rates are generally comparable to rates estimated for greater prairie chickens in other parts of their range, with a few exceptions. First, we estimated average hen survival for the 6‐month breeding season to be 0.68, greater than that reported for hens in Kansas over the same time span (0.45; Augustine & Sandercock, 2011). Assuming a mean overwinter survival rate of 0.93 (M.A. Hardy, *unpublished data*), hens in the CWGCA would have a mean annual survival rate of 0.63, similar to some estimates from Kansas (0.61; Winder et al., 2018), but notably greater than estimates of annual age‐specific survival observed in Wisconsin from 1950–1970 (0.24–0.57; Wisdom & Mills, 1997; calculated from composite life tables presented in Hamerstrom & Hamerstrom, 1973). Additionally, projecting over a 35‐day exposure period, our estimates of nest success (8.2%–28.6%) are slightly higher than nest success rates reported from Kansas (7.4%; Augustine & Sandercock, 2011) but comparable to estimates from Oklahoma (18.2%; Hovick et al., 2015), Nebraska (24.8%; Harrison et al., 2017), and the lower end of survival estimates reported from Missouri (28%–40%; McKee et al., 1998). Finally, our estimates of weekly brood survival at Buena Vista (0.88) and Paul Olson (0.89) are comparable to the lowest estimate of brood survival (0.88) presented by Wisdom and Mills (1997). However, projecting these rates over a 3‐week exposure period yielded estimates of 0.69, which are greater than survival rates reported for populations in the Nebraska Sandhills (0.59; Matthews et al., 2011) and the Flint Hills (0.27–0.29; McNew et al., 2012). Although it is difficult to attribute the differences we observed in this study to any single factor, we note that prairie chicken populations in central Wisconsin are relatively small and isolated with respect to most other populations across their range; thus, density‐dependent factors may play a role within local populations. Alternatively, our results may reflect geographic variation in vital rates due to different landscape composition or configuration compared to prairie chicken populations in the Great Plains. In any case, our findings underscore the value of investigating local, population‐level responses to land cover and management practices, as lessons learned elsewhere may not always be applicable across an entire species' range.

Here, we simultaneously examined multiple responses to land cover and management practices by conducting comprehensive demographic and habitat selection analyses for greater prairie chickens persisting in human‐modified grassland landscapes. Prairie chicken responses to different land cover classes and, especially, management practices, were highly variable among life‐history stages and suggest that a variety of management practices may be needed to accommodate prairie chickens throughout the annual cycle. For prairie chickens in the CWGCA, continued brush and tree removal may be especially beneficial: hens select for areas where this practice has occurred, with positive consequences for survival. However, we note that low to moderate amounts of shrub/tree cover appear to be beneficial for broods, so managers should take care to strike an appropriate balance in order to provide high‐quality habitat for both hens and broods. Likewise, prescribed fire provides attractive brood‐rearing habitat, but may compromise hen survival if too much of the landscape is burned. Finally, certain management practices associated with agricultural land use, such as hay cultivation and low‐ to moderate‐intensity grazing, may also benefit prairie chickens by providing concealed nest sites and high‐quality brood‐rearing habitat, but the timing of such practices is critical: Early harvest of hay fields may increase mortality of nests, chicks, and/or incubating hens. In conclusion, management regimes that promote heterogeneity in the vegetative structure of grassland systems can confer substantial benefits to numerous grassland bird species (Fuhlendorf et al., 2006; McNew et al., 2015; Rahmig et al., 2009; Winder et al., 2018), and in human‐modified landscapes such practices may well be the key to providing attractive, high‐quality habitat for grassland specialists during all life‐history stages.

## 5 Data Accessibility Statement

MARK input files, land cover and management practices at used and available points, and R code for running the resource selection function analyses have been deposited in the Dryad digital repository (https://doi.org/10.5061/dryad.dbrv15dzm).

## Conflict of interest

The authors have no conflicts of interest to declare.

## AUTHOR CONTRIBUTION

**Michael Hardy:** Conceptualization (equal); Data curation (equal); Formal analysis (lead); Methodology (equal); Writing‐original draft (lead); Writing‐review & editing (equal). **Matthew Broadway:** Conceptualization (equal); Data curation (equal); Investigation (equal); Methodology (equal); Writing‐review & editing (equal). **Christopher Pollentier:** Conceptualization (equal); Data curation (equal); Methodology (equal); Project administration (supporting); Writing‐review & editing (equal). **Volker Radeloff:** Conceptualization (equal); Methodology (equal); Writing‐review & editing (equal). **Jason Riddle:** Conceptualization (equal); Investigation (supporting); Methodology (equal); Project administration (supporting); Supervision (supporting); Writing‐review & editing (equal). **Scott Hull:** Conceptualization (equal); Funding acquisition (lead); Project administration (lead); Resources (lead); Supervision (lead); Writing‐review & editing (equal). **Benjamin Zuckerberg:** Conceptualization (equal); Formal analysis (supporting); Methodology (equal); Project administration (lead); Supervision (lead); Writing‐original draft (supporting); Writing‐review & editing (equal).

## Supporting information

Appendix S1

Appendix S2

Appendix S3
